# Using Decision Tree to Predict Cancer-Specific Mortality in Patients with Clear Cell Renal Cancer Treated with Nephrectomy

**DOI:** 10.3390/cancers18162644

**Published:** 2026-08-17

**Authors:** Laura Martínez-Cayuelas, Pau Sarrio-Sanz, Jose-Vicente Segura-Heras, Milagros Muñoz-Montoya, Vicente-Francisco Gil-Guillen, Jesus Romero-Maroto, Luis Gomez-Perez

**Affiliations:** 1 Urology Services, University Hospital of San Juan de Alicante, 03550 San Juan de Alicante, Alicante, Spain; 2 Instituto Centro de Investigación Operativa, Miguel Hernández University, 03202 Elche, Alicante, Spain; jvsh@umh.es; 3 Clinical Medicine Department, Miguel Hernández University, 03550 San Juan de Alicante, Alicante, Spain; 4 Pathology and Surgery Department, Miguel Hernández University, 03550 San Juan de Alicante, Alicante, Spainl.gomez@umh.es (L.G.-P.); 5 Urology Services, University and General Hospital of Elche, 03203 Elche, Alicante, Spain

**Keywords:** kidney cancer, mortality, predictions and projections, decision trees

## Abstract

**Background/Objectives**: Accurate prognostic stratification after nephrectomy for clear cell renal carcinoma (ccRCC) remains challenging. Traditional models often lack the intuitive clinical application or the ability to handle non-linear interactions between variables. We aimed to develop and internally validate a decision tree-based model to predict cancer-specific survival in patients with ccRCC following nephrectomy. **Methods**: We analyzed 79,526 patients with ccRCC who underwent nephrectomy from the SEER database (2012–2018). Patients were randomized into development (2/3) and validation (1/3) cohorts. A conditional inference tree was constructed to predict cancer-specific survival. Multiple imputation by chained equations was used to handle missing data. Discriminatory ability was assessed using the C-index. Net clinical benefit was evaluated with decision curve analysis. The model was evaluated using CHARMS and PROBAST. **Results**: A decision tree with 15 risk groups is presented, further classified into high-, intermediate-, and low-risk categories according to observed median survival. The final predictors were tumor localization, tumor grade, TNM stage, age, and sarcomatoid differentiation. The model demonstrated excellent discriminatory performance, with a C-index of 0.846 (95% CI: 0.834–0.847). PROBAST assessment showed low risk of bias and low concern regarding applicability. **Conclusions**: The use of decision trees provides an interpretable alternative to conventional regression-based models. Three main risk categories and 15 subgroups are proposed based on tumor localization, tumor grade, TNM stage, age, and sarcomatoid differentiation. Our model demonstrates good applicability and a low risk of bias according to PROBAST guidelines; however, external validation in independent cohorts is required prior to clinical implementation.

## Simple Summary

Traditional prognostic models for renal cell carcinoma may be difficult to apply in routine practice and may not adequately capture non-linear relationships between clinical and pathological variables. We developed and internally validated a decision tree-based model using data from 79,526 patients with clear cell renal cell carcinoma treated with nephrectomy. The model identified 15 prognostic subgroups, which were further grouped into three risk categories. It showed strong discrimination in the validation cohort (Harrell’s C-index: 0.835) and a low risk of bias, providing an intuitive framework for postoperative risk stratification.

## 1. Introduction

Renal cell carcinoma accounts for approximately 2% of all cancer-related deaths worldwide in developed countries and is among the ten most common malignancies. The clear cell subtype, in particular, is associated with the poorest prognosis [1]. Its incidence has increased by approximately 2% both in Europe and globally, with an estimated incidence rate of 12 new cases per 100,000 individuals [2].

The prognosis of clear cell renal cell carcinoma (ccRCC) is highly variable and depends largely on the clinical stage at diagnosis. Overall survival rates have improved over time, likely due to the incidental detection of these tumors and a consequent increase in the diagnosis of early-stage, localized disease [3].

Survival is significantly poorer in metastatic cases, with a 5-year survival rate of only 14% [4,5]. Therefore, accurate risk stratification is essential to identify patients at higher risk of developing metastases and to enable early detection. Prognostic models play a key role in guiding individualized patient follow-up.

Despite the proliferation of prognostic tools, their clinical integration remains suboptimal. Our recent systematic review [6] of 40 models using the CHARMS [7] and PROBAST [8] frameworks revealed a sobering reality: 100% of the models exhibited a high risk of bias, and only 40% demonstrated adequate clinical applicability. A primary limitation stems from the widespread reliance on Cox proportional hazards models. While statistically powerful, Cox models often fail to capture complex, non-linear interactions between clinicopathological variables and require strict adherence to the proportional hazards assumption, which is frequently violated in long-term oncological data.

The aim of our study is to develop and internally validate a decision tree for cancer-specific survival (CSS) in ccRCC, addressing the statistical limitations of previous models and adhering to the CHARMS and PROBAST guidelines.

## 2. Materials and Methods

### 2.1. Patients

Data from surgically treated ccRCC patients were obtained from the Surveillance, Epidemiology, and End Results Program (SEER) database between 2012 and 2018. This database contains anonymized, population-based cancer data collected from regional registries and covers approximately 26.5% of the US population. Institutional Review Board approval was obtained (AUT.DMC.LMC.251020).

Inclusion criteria were as follows: diagnosis of renal cancer (C649) with clear cell histology (codes 8310 and 8312) treated with total or partial nephrectomy (codes 30, 40, 50, 70, and 80).

The dataset was extracted, including the following variables: age, sex, year of diagnosis, race, survival months, SEER summary stage, TNM stage, tumor size, nuclear grade (I, II, III, and IV), and presence of sarcomatoid features.

The survival months variable represents the time each patient survived from the time of RCC diagnosis. The SEER cause-specific death classification variable was used as the censoring variable, indicating whether the event occurred, with patients classified as alive, dead from ccRCC, or dead from other causes.

The T stage was simplified into T1, T2, T3, and T4 categories. The N and M stages were dichotomized as positive or negative.

### 2.2. Statistical Analysis

Categorical variables were described using frequencies and percentages. Continuous variables were treated as linear and summarized using means and standard deviations. For model development, the dataset was randomly split into a development cohort (2/3 of the total sample) and a validation cohort (remaining 1/3).

The first step consisted of comparing the two cohorts using Pearson’s chi-square test or analysis of variance (ANOVA), depending on the type of variable. The assumption of normality was assessed using the Kolmogorov–Smirnov–Lilliefors test.

**Figure 1:**
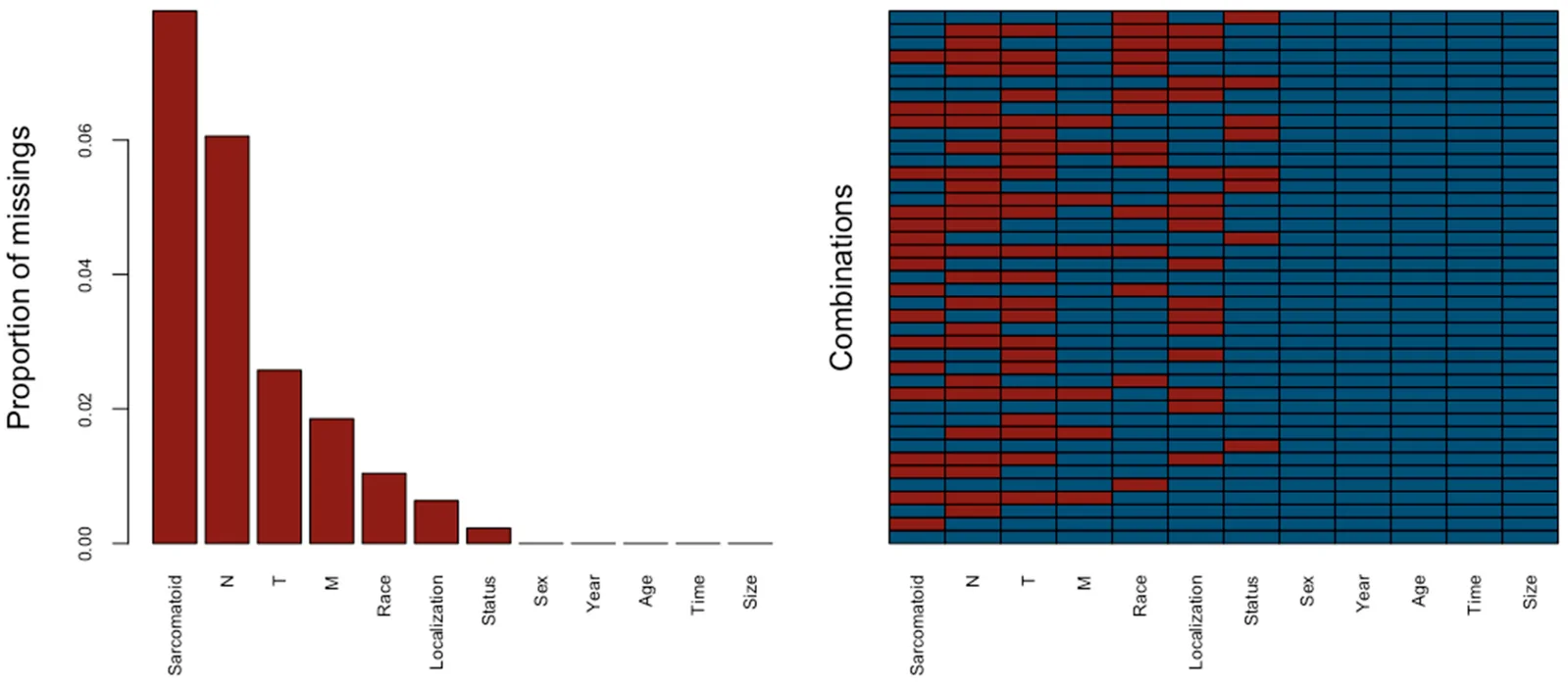
Proportion and patterns of missing data across variables.

Missing data were handled using multiple imputation by chained equations (MICE), applying classification trees for categorical variables and regression trees for continuous variables. This process involved sequentially imputing missing values based on fully conditional specification models appropriate for each type of risk factor, using all other available covariates along with the outcome. Five multiple imputations were performed using the CART method. Figure 1 shows the proportion of missing data for each variable.

Conditional inference classification trees were constructed to improve upon the performance of traditional Cox proportional hazards models. These trees are based on conditional inference for survival analysis with censored data, without assuming proportional hazards, and allow flexibility to model different survival curves across patient subgroups. Regression relationships were estimated through recursive binary partitioning within a conditional inference framework, ensuring appropriate tree growth without the need for additional cross-validation procedures [9].

Model performance was assessed by quantifying discrimination using Harrell’s concordance index (C-index). Net benefit was evaluated with decision curve analysis (DCA). All statistical analyses were performed using R statistical software (version 4.5.0). A two-sided *p*-value < 0.05 was considered statistically significant.

Calibration at 36 months was assessed in the validation cohort by comparing predicted survival probabilities with observed survival estimated using the Kaplan–Meier method. Patients were grouped according to predicted risk, and calibration plots were generated against the line of perfect agreement, with point sizes reflecting group sample size.

### 2.3. Model Evaluation

Finally, a blinded author assessed the risk of bias and applicability using the PROBAST tool and extracted the relevant items of the model following the CHARMS guidelines, in order to identify potential sources of bias and summarize the model characteristics [7,8,10].

## 3. Results

A total of 79,526 patients with ccRCC treated surgically with total or partial nephrectomy were included. Of these, 53,017 patients (2/3 of the total sample) were assigned to the development cohort and 26,509 to the validation cohort (remaining 1/3).

Table 1 summarizes the sociodemographic characteristics of the overall population, as well as those of each cohort, including the comparison between them. Only in sarcomatoid differentiation statistically significant differences were observed between the two cohorts (*p* > 0.05).

**Figure 2:**
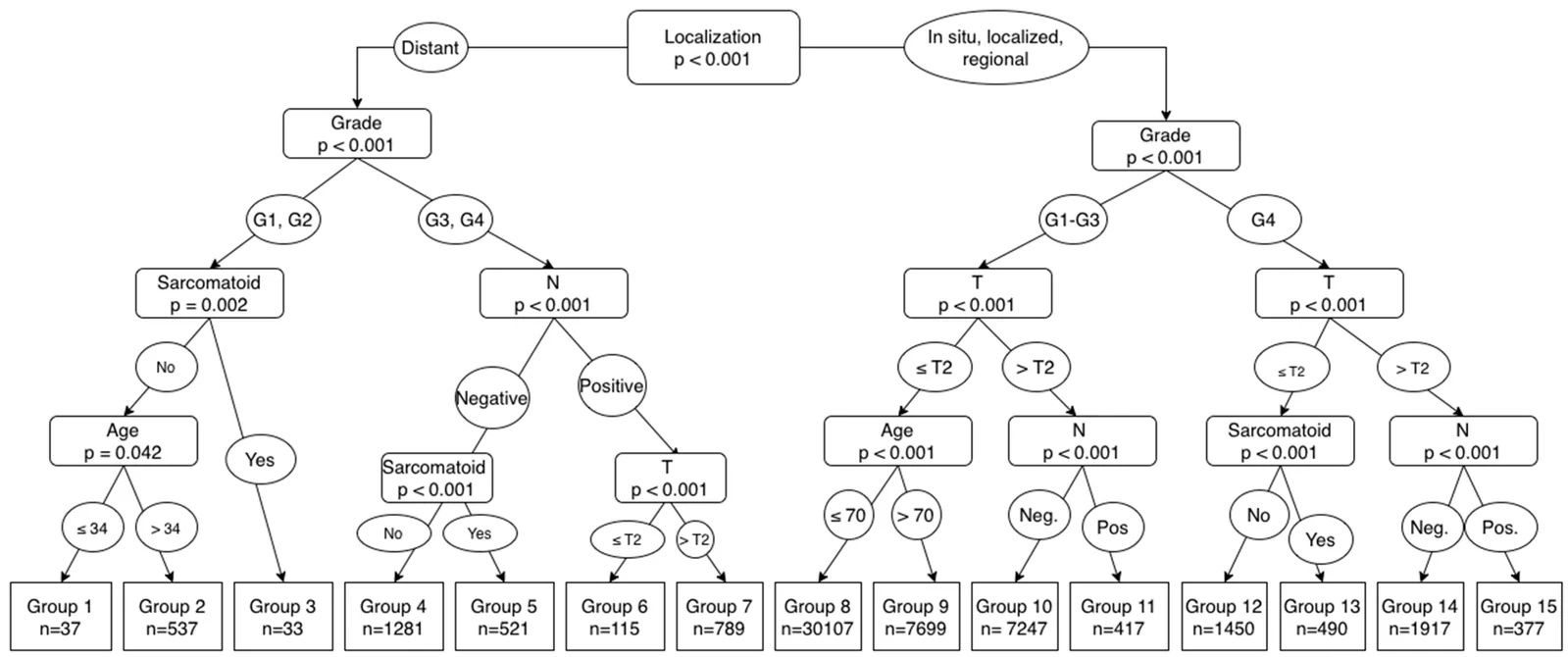
Conditional inference trees.

We identified 15 risk groups with significant differences in cancer-specific survival (*p* < 0.01). The final conditional inference tree comprised 15 terminal nodes generated by 14 binary splits, reflecting the effective complexity of the model. Figure 2 shows the decision tree in which the variable with the greatest weight for classifying patients into different groups is “Localization”. Accordingly, patients with distant involvement (metastatic disease) have poorer prognosis than those without. Other variables included in the tree, due to their statistical significance in discriminating among subgroups, are tumor grade, TNM stage, age, and sarcomatoid differentiation.

Table 2 shows the events and median survival for each group. The nodes were further consolidated into three clinical risk categories based on survival probability. The subgroups with the poorest prognosis are groups 3, 4, 5, 6, 7, and 15, with a median survival of less than 40 months; these were classified as high risk. The subgroups with the best prognosis are groups 1, 8, 9, 10, and 12, with a median survival of more than 70 months; these were classified as low risk. The remaining subgroups (groups 2, 11, 13, and 14) have a median survival of between 40 and 70 months and were classified as intermediate risk.

Supplementary Material S1 presents the Kaplan–Meier survival curves for each subgroup in the derivation and validation cohorts, together with the corresponding log-rank test *p*-values.

**Figure 3:**
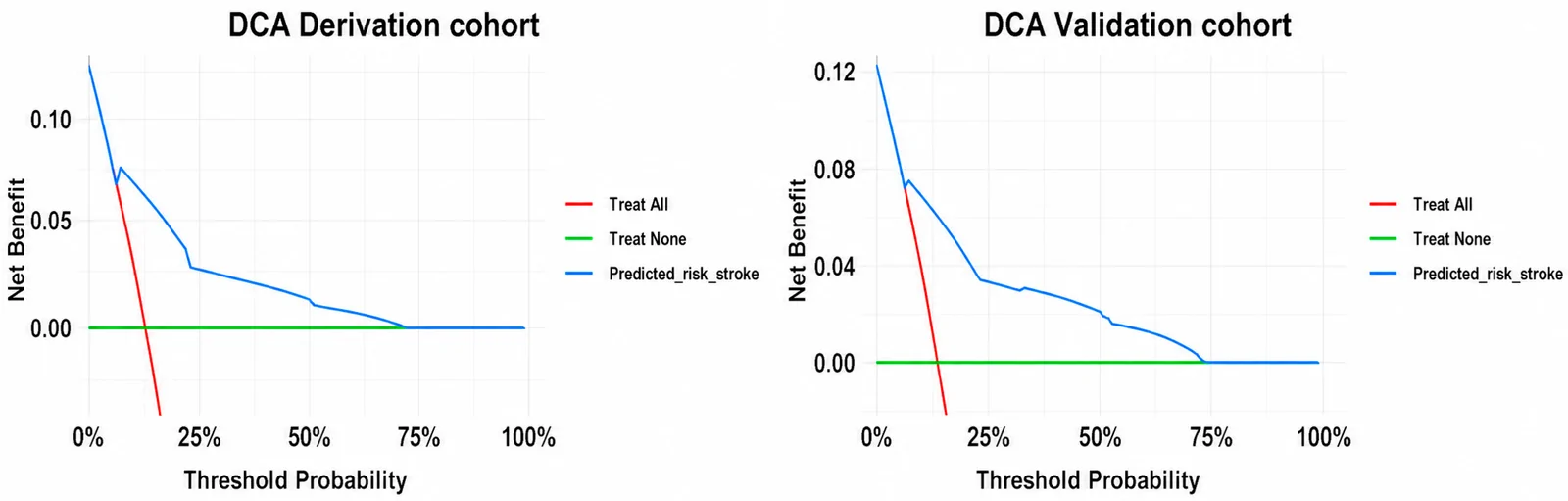
Decision curve Analysis.

Decision curve analysis (DCA, Figure 3) demonstrated that the classification tree model provides greater net clinical benefit than the “treat all” or “treat none” strategies across a wide range of threshold probabilities (approximately 5–73%) in both the derivation and validation cohorts. This suggests that the algorithm is beneficial for clinical decision-making without disproportionately increasing false positives.

The model demonstrated excellent discriminative ability, with a C-index of 0.846 (95% CI: 0.834–0.847) in the development cohort. This accuracy remained robust in the internal validation cohort, with a C-index of 0.835 (95% CI: 0.818–0.838), confirming the stability of the survival tree for risk stratification.

**Figure 4:**
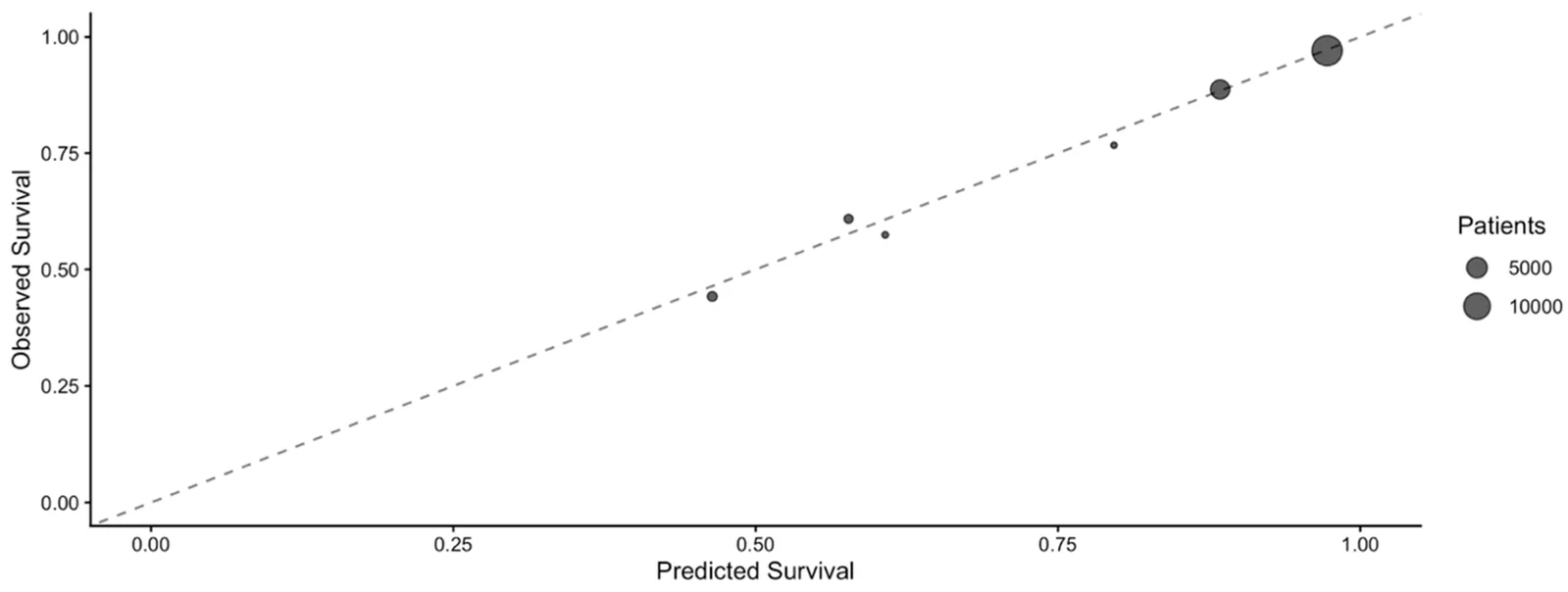
Calibration plot at 36 months for validation cohort. The size of each circle was proportional to the sample size included in each subgroup.

The model also showed strong calibration across the full risk spectrum at 36 months. The predicted survival probabilities were consistent with the observed outcomes, with most points closely aligned with the diagonal line (Figure 4).

A descriptive table was created using the CHARMS items (Table 3). The model demonstrated a low risk of bias, as well as good applicability according to the PROBAST criteria (Supplementary Material S2).

## 4. Discussion

The proposed model develops a conditional decision tree for patients with ccRCC undergoing nephrectomy, using the SEER database between 2012 and 2018. A total of 79,526 subjects were included, and internal validation was performed using one third of the overall sample.

Fifteen risk groups were identified based on the variables described above: tumor localization, tumor grade, TNM stage, age, and sarcomatoid differentiation. Median survival across groups ranged from 81.3 months in the best prognosis group (subgroup 8) to 23.6 months in the worst prognosis group (subgroup 7). To simplify stratification in our decision tree, subgroups can be classified into three major risk categories: high, intermediate, and low.

Previous studies have applied tree-based and other machine-learning approaches to prognostic prediction in RCC. Jiang et al. compared decision trees with several machine-learning algorithms for predicting overall survival in a large SEER cohort, while Zhang et al. evaluated decision tree and ensemble models for predicting three-year survival and metastasis in patients with kidney cancer [11,12]. However, these studies included different RCC histological subtypes and treated survival as a fixed-time classification outcome. In contrast, the present study specifically evaluated cancer-specific survival in patients with ccRCC undergoing nephrectomy using a conditional inference tree designed for censored survival data.

In our systematic review of predictive models for renal cancer [6] we identified a high risk of bias and low applicability after evaluation using CHARMS and PROBAST tools. All included models (100%) were based on logistic regression, a method associated with a higher risk of error due to the fact that its proportional hazards assumption may not always hold in practice [9,13,14]. In this context, we developed a decision tree-based model using conditional inference trees, a more flexible approach for survival estimation that has shown promising results in pathologies such as bladder [13] and breast cancer [9]. However, the present study was not designed to demonstrate superiority over established ccRCC prognostic models.

All of the variables in the decision tree have been used in prognostic models for renal cancer reported in the literature [6].

The prognosis of ccRCC is determined primarily by SEER summary stage (localized, regional, or distant). Patients presenting with distant disease have poorer median survival, in line with the findings reported in previous nomograms [15].

TNM staging is fundamental in the general classification of tumors and is therefore one of the most important variables in the construction of the decision tree. It was used in 62–70% of the models reviewed [6] and in all cases was among the variables with the greatest weight in the proposed nomograms, as seen in studies by Tian et al. and Xiao et al. [16,17]. The model proposed by Kanao et al. includes only TNM stage, highlighting the strong predictive value of this variable, which alone provides robustness to the model, although it has been criticized for a lack of precision as it excludes other relevant variables [18]. Although metastasis type was not analyzed in our study, it has been addressed in other publications; for example, Cho et al. [19] demonstrated that the presence of liver metastases is linked to poorer prognosis than other metastatic sites.

T stage has been shown to be one of the most important prognostic factors in nomograms developed for non-metastatic tumors, with higher T stages associated with poorer outcomes [20]. Studies such as that by Su et al. [21] in papillary RCC found that T stage is the variable with the greatest prognostic weight, with a higher risk of cancer-specific mortality in patients with more advanced T stages [18]. Tumor size has also been identified as a key factor; according to Karakiewicz et al. [22], larger tumors are associated with greater aggressiveness and poorer prognosis.

Tumor grade is another important variable clearly linked to prognosis, particularly at grades 3 and 4, which are associated with more aggressive tumors. When combined with distant disease, it classifies patients into high- and intermediate-risk groups. Grade was included in 51% of cases in previous models [6] and also carries substantial weight in prognostic models described in the literature [16,22].

Although tumor size is a well-established prognostic factor in ccRCC and was included among the candidate predictors, it was not included in the final conditional inference tree [23]. This may be explained by the substantial predictive overlap between tumor size and T stage, as tumor size is an important component of the T classification, particularly in organ-confined disease.

Age is associated with the development of renal cancer, with diagnosis occurring most frequently in the sixth and seventh decades of life; it is relatively uncommon below the age of 40, although it may occur in the context of hereditary syndromes [24,25]. Age is an important variable and is therefore included in the decision tree; however, it has less impact than the variables described above. Age above 70 years is associated with poorer prognosis. In previous studies, age was included in 48% of models [6]. Articles such as Tang et al. [20] suggest that age plays a crucial role in the prognosis of renal cancer. Increasing age affects the immune system, which may contribute to tumor progression and a consequent reduction in survival [15,20]. In series such as Tian et al. [16], which focus on young patients (<46 years), individuals diagnosed between 39 and 46 years showed poorer prognosis than those diagnosed under 39, likely related to a greater accumulation of genetic mutations and the development of more aggressive tumors within this age range.

In contrast to our findings, Su et al. [21], in the context of papillary RCC, reported that increasing age was associated with higher overall mortality from other causes, without the rise in cancer-specific mortality observed in our data. This discrepancy may be explained by the intrinsic differences between papillary and clear cell tumors, as the former typically occur in older individuals with associated comorbidities and tend to be less aggressive. Consequently, these patients are more likely to die from causes other than cancer.

Finally, sarcomatoid differentiation does not carry as much weight as TNM stage or tumor grade; however, it is associated with poorer prognosis in the final classifications. Only 13% of published models included it as a predictor [6]. In the study by Leibovich et al. [26], it was associated with nearly a twofold increase in mortality risk, while in the study by Cho et al. [19], it was identified as the factor with the greatest impact.

The main strength of our model is the large number of patients included, made possible by the SEER database, which provides substantial statistical power. Additionally, the use of a validated methodology and its critical appraisal using PROBAST ensure a low risk of bias and good applicability. Furthermore, the model is readily applicable in real-world clinical practice, as data for all included variables are routinely available from medical records and standard diagnostic tests.

Regarding limitations, this model was developed exclusively for patients with ccRCC and should not be extrapolated to other RCC histological subtypes. In addition, the model does not include patient-related baseline variables (such as weight loss, fever, or asthenia), or tumor-related biomarkers such as PD-L1 expression, as these data are not available in the SEER database [27]. Previous studies have shown that constitutional symptoms [19,22,26] and laboratory abnormalities may influence prognosis and survival outcomes in RCC patients [28]. Therefore, future studies incorporating host-related clinical and laboratory factors may improve prognostic accuracy. Although sarcomatoid differentiation showed a statistically significant difference, this may partly reflect the large sample size rather than a clinically meaningful imbalance.

The absence of external validation remains a limitation of the present study. Although split-sample internal validation demonstrated stable discrimination, this does not establish model transportability to populations outside the SEER database. External validation should therefore be performed in independent multicenter cohorts from different geographical regions and healthcare systems, following the methodological framework proposed by Debray et al. [29,30]. Missing data and loss to follow-up may have introduced selection bias, although this was partially mitigated through mathematical imputation methods. Finally, despite internal validation during model development, external validation in independent populations and different geographic regions is required before clinical implementation.

## 5. Conclusions

Clear cell RCC is a heterogeneous disease with a variable prognosis depending on its presentation. We propose a decision tree-based model that addresses some of the statistical limitations of previous models. Fifteen subgroups were defined and subsequently classified into three main risk categories based on tumor localization, tumor grade, TNM stage, age, and sarcomatoid differentiation.

Our model demonstrates good applicability and a low risk of bias according to the PROBAST guidelines; however, external validation in independent cohorts is required prior to its use in clinical practice.

## Figures and Tables

**Table 1:** Sociodemographic characteristics of the patients included in the model.

	Overall N = 79,526 ^1^	Derivation N = 53,017 ^1^	Validation N = 26,509 ^1^	*p*-Value ^2^
**Sex**				0.831
Female	28,956 (36.4%)	19,289 (36.4%)	9667 (36.5%)	
Male	50,570 (63.6%)	33,728 (63.6%)	16,842 (63.5%)	
**Year**				0.801
2012	10,665 (13.4%)	7116 (13.4%)	3549 (13.4%)	
2013	10,742 (13.5%)	7134 (13.5%)	3608 (13.6%)	
2014	11,051 (13.9%)	7403 (14%)	3648 (13.8%)	
2015	11,493 (14.5%)	7666 (14.5%)	3827 (14.4%)	
2016	11,657 (14.7%)	7703 (14.5%)	3954 (14.9%)	
2017	12,139 (15.3%)	8106 (15.3%)	4033 (15.2%)	
2018	11,779 (14.8%)	7889 (14.9%)	3890 (14.7%)	
**Age (years)**	60.38 (13.74)	60.36 (13.77)	60.43 (13.68)	0.529
**Race**				0.403
American Indian	819 (1.0%)	565 (1.1%)	260 (1.0%)	
Asian or Pacific Islander	4604 (5.8%)	3078 (5.8%)	1526 (5.8%)	
Black	9571 (12.0%)	6436 (12.1%)	3135 (11.8%)	
White	64,532 (81.1%)	42,944 (81.0%)	21,588 (81.4%)	
**Status**				0.267
Alive or dead from other cause	72,986 (91.8%)	48,617 (91.7%)	24,369 (91.9%)	
Dead (from RCC)	6540 (8.2%)	4400 (8.3%)	2140 (8.1%)	
**Survival time (months)**	34.78 (24.04)	34.76 (24.05)	34.81 (24.01)	0.799
**Summary stage**				0.602
In situ	6 (0.0%)	5 (0.0%)	1 (0.0%)	
Localized	59,301 (74.6%)	39,474 (74.5%)	19,827 (74.8%)	
Regional	15,275 (19.2%)	10,227 (19.3%)	5048 (19.0%)	
Distant	4944 (6.2%)	3313 (6.2%)	1631 (6.2%)	
**T stage**				0.258
T1	52,628 (66.2%)	35,058 (66.1%)	17,570 (66.3%)	
T2	8283 (10.4%)	5473 (10.3%)	2810 (10.6%)	
T3	17,421 (21.9%)	11,673 (22.0%)	5748 (21.7%)	
T4	1189 (1.5%)	811 (1.5%)	378 (1.4%)	
**N stage**				0.440
Negative	76,456 (96.1%)	50,950 (96.1%)	25,506 (96.2%)	
Positive	3070 (3.9%)	2067 (3.9%)	1003 (3.8%)	
**M stage**				0.739
Negative	74,689 (93.9%)	49,781 (93.9%)	24,908 (94.0%)	
Positive	4837 (6.1%)	3236 (6.1%)	1601 (6.0%)	
**Size (millimeters)**	64.18 (111.81)	63.74 (110.38)	65.06 (114.61)	0.116
**Sarcomatoid differentiation**	3725 (4.7%)	2570 (4.8%)	1155 (4,4%)	0.003
**Grade**				0.069
G1	8307 (10.4%)	5501 (10.4%)	2806 (10.6%)	
G2	39,367 (49.5%)	26,300 (49.6%)	13,067 (49.3%)	
G3	23,498 (29.5%)	15,562 (29.4%)	7936 (29.9%)	
G4	8354 (10.5%)	5654 (10.7%)	2700 (10.2%)	

^1^ n (%); mean (SD). ^2^ Pearson’s chi-squared test with simulated *p*-value (based on 2000 replicates). Two-sample *t*-test.

**Table 2:** The number of patients included in each subgroup, along with the number of events, median survival, and the corresponding 95% confidence intervals. Open intervals have been labeled as “Not Estimable” (NE).

Group	*n*	Events	r Mean (SE)	Median Survival	95% CI
1	37	0	81.0 (0.0)		
2	537	165	57.0 (1.6)		70.0–NE
3	33	19	26.4 (3.7)	22	16.0–NE
4	1281	565	44.7 (1.1)	38	34.0–43.0
5	521	305	30.6 (1.6)	17.0	14.0–22.0
6	115	51	44.7 (3.7)	36	30.0–NE
7	789	533	23.6 (1.1)	13.0	11.0–14.0
8	30,107	573	81.3 (0.1)		
9	7699	417	77.9 (0.2)		
10	7247	697	73.9 (0.3)		
11	417	148	49.7 (1.9)	54.0	42.0–NE
12	1450	141	74.4 (0.7)		
13	490	100	62.4 (1.8)		
14	1917	488	59.7 (0.9)		
15	377	198	37.8 (1.9)	25	22.0–32.0

**Table 3:** Relevant items extracted from the included decision tree model for predicting cancer-specific survival in renal cancer, based on the Critical Appraisal and Data Extraction for Systematic Reviews of Prediction Modelling Studies (CHARMS) checklist. The denominator of 20 used in the initial EPV calculation represented the total number of candidate predictor terms after accounting for the categories of the categorical predictors.

CHARMS Items	
	Martínez-Cayuelas et al., 2025 [6]
Source of data	Retrospective cohort study
Participants	SEER database Consecutive inclusion of patients in two cohorts resulting in 2/3 (53,017 patients) and 1/3 (26,509 patients) in the validation group Patients with renal cancer treated with nephrectomy Treatment is not included as a candidate predictor Baseline data: 2012–2018
Outcome to be predicted	Cancer specific survival Measurement: medical records Blinding unknown
Candidate predictors	Predictors: sex, age, race, SEER cause-specific death classification, survival time, TNM stage, tumor size, tumor grade, and sarcomatoid differentiation Measurement: clinical records at baseline (at diagnosis) Blinding of measurement is unknown, although all variables are objective Continuous predictors: linear
Sample size	N = 53,017 (E = 4400) to develop the model and 26,509 to validate it (E = 2140) EPV to develop the model: 4400/20 = 220 EPV internal validation: 2140/20 = 107
Missing data	Multiple imputation by chained equations (MICE)
Model development	Conditional inference trees for survival analysis Assumptions were not tested Method for selecting predictors for inclusion in multivariable modeling: full model Method for selecting predictors during multivariable modeling: full model No shrinkage or penalization
Model performance	Discrimination: C-index Calibration: calibration plots Classification measures: none
Model evaluation	Decision curve analysis (DCA)
Results	Indicated: model coefficients, c-index, DCA, and calibration plot Not indicated: none Presentation: conditional classification trees Comparison of the distribution of the predictors for development and validation data sets
Interpretation and discussion	Exploratory results Comparison with previous models and explanation for the predictors of the final models Analysis of strengths and limitations Discussion of generalizability in other areas Acknowledgement of the need for external validation

## Data Availability

The original contributions presented in this study are included in the article/Supplementary Materials. Further inquiries can be directed to the corresponding author.

## Supplementary Materials

The following supporting information can be downloaded at https://www.mdpi.com/article/10.3390/cancers18162644/s1. Supplemental Material S1. Kaplan–Meier survival curves for each subgroup in the derivation and validation cohorts, together with the corresponding log-rank test *p*-values. Supplemental Material S2: Checklist for the evaluation of the model developed according to Prediction model study Risk Of Bias Assessment Tool.

## Abbreviations

The following abbreviations are used in this manuscript:

ccRCC	Clear Cell Renal Cell Carcinoma
CSS	Cancer-Specific Survival
SEER	Surveillance, Epidemiology, and End Results Program
ANOVA	Analysis of Variance
MICE	Multiple Imputation by Chained Equations
DCA	Decision Curve Analysis
