# Supplementary material for: Using Decision Tree to Predict Cancer-Specific Mortality in Patients with Clear Cell Renal Cancer Treated with Nephrectomy
Source: Cancers (Basel). 2026 Aug 17;18(16):2644. doi: 10.3390/cancers18162644 (PMC13510597; doi:10.3390/cancers18162644)
Supplement: Supplementary file 1 [file cancers-18-02644-s001.zip › Supplementary Material S1.pdf]

Supplementary Material S1.

Kaplan–Meier survival curves for each subgroup in the derivation and validation cohorts, together with the corresponding log-rank test p-values.

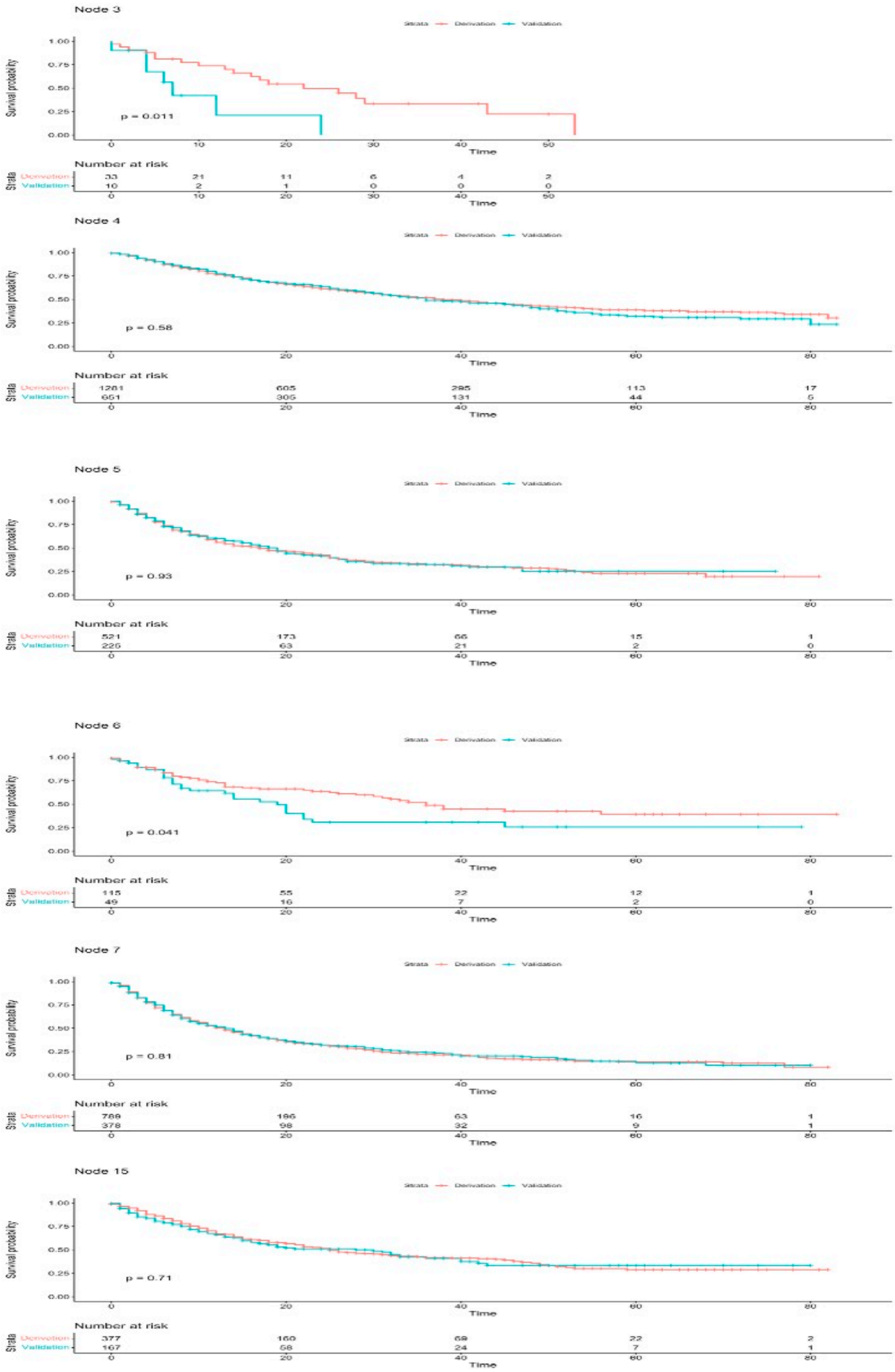

1 High-risk groups survival curves

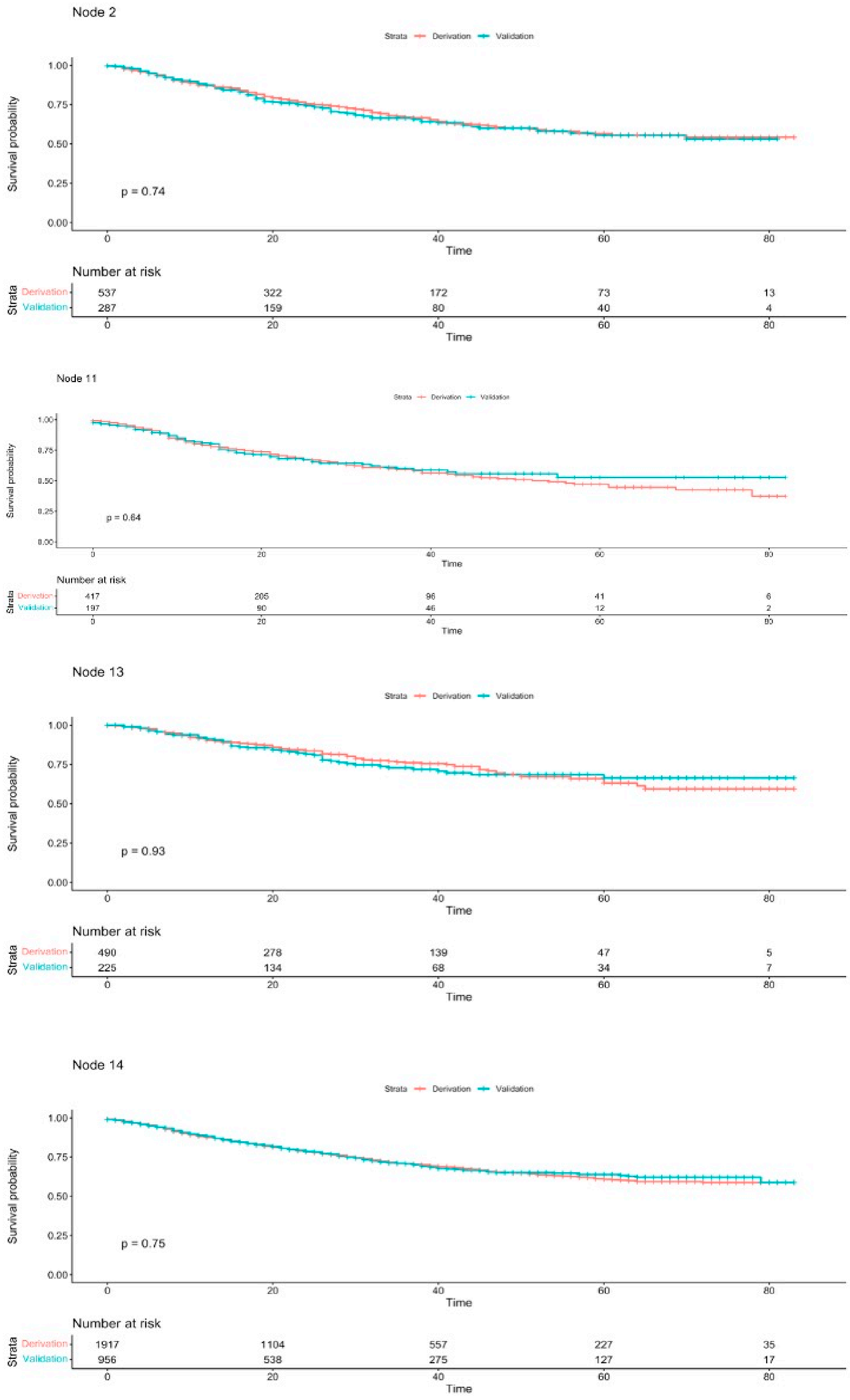

2. Intermediate-risk groups survival curves

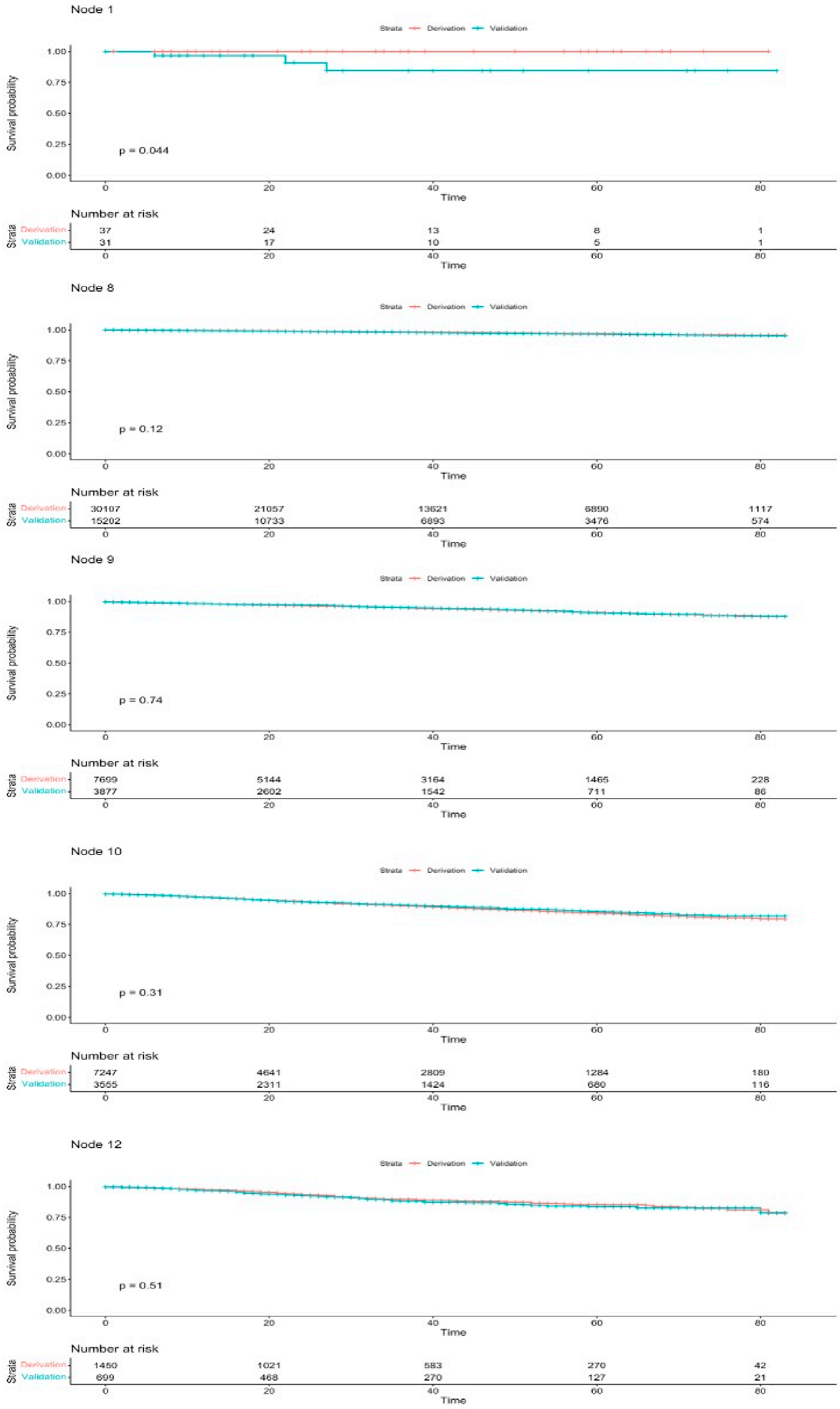

3 Low-risk groups survival curves
